# Curcumin nanoparticles alleviate brain mitochondrial dysfunction and cellular senescence in γ-irradiated rats

**DOI:** 10.1038/s41598-025-87635-y

**Published:** 2025-01-31

**Authors:** Omnia A. Moselhy, Nahed Abdel-Aziz, Azza El-bahkery, Said S. Moselhy, Ehab A. Ibrahim

**Affiliations:** 1 Biochemistry Department, Faculty of Science, Ain Shams University, Cairo, Egypt; 2 Radiation Biology Research Department, National Center for Radiation Research & Technology, Egyptian Atomic Energy Authority, Cairo, Egypt

**Keywords:** Mitochondrial dysfunction, Cellular senescence, Β-galactosidase, AMPK, p53-p21/p12 signaling pathway, Biochemistry, Physiology

## Abstract

Despite the diverse applications of γ radiation in radiotherapy, industrial processes, and sterilization, it causes hazardous effects on living organisms, such as cellular senescence, persistent cell cycle arrest, and mitochondrial dysfunction. This study evaluated the efficacy of curcumin nanoparticles (CNPs) in mitigating mitochondrial dysfunction and cellular senescence induced by γ radiation in rat brain tissues. Four groups of male Wistar albino rats (*n* = 8 per group) were included: (Gr1) the control group; (Gr2) the CNPs group (healthy rats receiving oral administration of curcumin nanoparticles at a dose of 10 mg/kg/day, three times per week for eight weeks); (Gr3) the irradiated group (rats exposed to a single dose of 10 Gy head γ irradiation); and (Gr4) the irradiated + CNPs group (irradiated rats treated with CNPs). The data obtained demonstrated that oral administration of CNPs for eight weeks attenuated oxidative stress in γ-irradiated rats by lowering the brain’s lipid peroxidation level [malondialdehyde (MDA)] and enhancing antioxidant markers [superoxide dismutase (SOD), reduced glutathione (GSH), and total antioxidant capacity (TAC)] (*P* < 0.05). In addition, CNPs significantly increased mitochondrial function by improving complex I, complex II, and ATP production levels compared to the irradiated group. In irradiated rats, CNPs also showed anti-neuroinflammatory effects by reducing brain interleukin 6 (IL-6), tumor necrosis factor-alpha (TNF-α), and nuclear factor-kappa B (NF-ĸB) levels (*P* < 0.05). Moreover, CNPs administered to irradiated rats significantly reduced brain β-galactosidase activity and the expression levels of p53, p21, and p16 genes (*P* < 0.05) while concurrently inducing a significant increase in AMPK mRNA expression compared to the irradiated group. In conclusion, CNPs ameliorated the neurotoxicity of γ radiation and hold promise as a novel agent to delay cellular senescence via their combined antioxidant, anti-inflammatory, and mitochondrial-enhancing properties.

## Introduction

Cellular senescence is a natural physiological process associated with aging. It is characterized in most cases by an irreversible cell cycle arrest in response to endogenous and exogenous stress stimuli, resistance to apoptosis, and a senescence-associated secretory phenotype (SASP)^1^. Cellular senescence is critical in determining cell fate and is essential for growth regulation, cancer prevention, and wound healing. However, chronic inflammation and persistent oxidative stress can lead to cellular senescence when cells are exposed to prolonged sub-lethal injury, as observed during chemotherapy or radiation therapy^2^.

Mitochondrial and metabolic dysfunction, telomere shortening, dysregulated autophagy, oxidative stress, and systemic inflammation contribute to cell cycle arrest during senescence. Among these factors, senescence-associated mitochondrial dysfunction is the most critical^3,4^. Senescent cells secrete proteases, growth factors, and inflammatory cytokines (e.g., interleukin-6 and interleukin-8), collectively called the senescence-associated secretory phenotype (SASP). They also produce excessive reactive oxygen species (ROS) and alter the microenvironment, promoting tumor development. Persistent cellular senescence can accelerate aging by impairing tissue regeneration, repair, and renewal^5^.

Senescence and malfunction of the mitochondria are two closely related characteristics of aging^3^. In aged tissues and senescent cells, mitochondrial dysfunction reduces each mitochondrion’s steady-state membrane potential and respiration capability^6^. Moreover, mitochondrial dysfunction causes severe impairment in cellular energy transformation, particularly in tissues highly dependent on the chemical energy produced by mitochondria for survival. Therefore, alterations in mitochondrial oxidative phosphorylation, which ultimately lead to mitochondrial dysfunction via elevated reactive oxygen species (ROS) byproducts, may result in age-related metabolic disorders^7^. Subsequently, elevated ROS can lead to increased inflammation and oxidative stress, promoting mitochondrial dysfunction and apoptosis^8^. Thus, enhancing mitochondrial function is a potential therapeutic target for promoting cell activity and preventing cell senescence.

Senescent cells can accelerate the aging process in an organism. As individuals age, they often experience a pro-inflammatory state, characterized by elevated levels of inflammatory mediators in the blood^9^. This state increases the risk of developing chronic age-associated disorders, including certain cancers, neurological diseases, and cardiovascular conditions. Additionally, inflammatory substances contribute to weight loss, muscular atrophy, depression, and senescent cells in older people. Collectively, these factors may inhibit progenitor and stem cell proliferation and prevent tissue regeneration^10^.

Recently, γ-radiation has been widely utilized in various fields, including medicine for therapeutic purposes, food sterilization, and as a fuel source. Its antiproliferative effects are particularly valuable in targeting and destroying cancer cells^11^. Nevertheless, γ-radiation can exert side effects such as oxidation of cellular proteins, DNA damage, ROS formation, lipid peroxidation, and inflammation^12^. Furthermore, γ-radiation emitted from an explosion a few kilometers away can significantly reduce the Earth’s ozone layer and increase the quantity of ultraviolet radiation reaching the Earth’s surface^13^.

In recent years, there has been a growing interest in research on radiation-induced cellular senescence, as people are increasingly exposed to radiation in many aspects of their daily lives^14^. Neurocognitive, neurosensory, and neurological impairments; musculoskeletal growth impairment; cardiovascular, cerebrovascular, endocrine, gastrointestinal, gonadal, reproductive, hepatic, pulmonary, and urinary systems are among the adverse outcomes of radiation exposure^15^. In elderly humans and animals, senescent cells can accumulate due to acute stresses, such as radiation^16^. Therefore, we employed γ radiation in a rat model to accelerate the aging process and induce the development of senescent cells.

There is no specific marker for the diagnosis and prognosis of senescence^17^. This is partially due to the diversity and heterogeneity of tissues and the differences in how each one reacts to the wide range of genotoxic stimuli. Identifying a panel of distinct markers based on cell cycle arrest (such as p35, p21, and p16), increased lysosomal compartment activity (senescence-associated β-galactosidase), structural alterations linked to the DNA damage response, and additional features unique to the senescence-associated secretory phenotype (SASP), such as increases in ROS and pro-inflammatory cytokines/chemokines, is among the most common approaches^1,18^.

AMPK (AMP-activated protein kinase) is a critical regulator of cellular energy homeostasis. It detects low energy levels in the cell and activates pathways to restore balance. Its activation promotes autophagy, reduces oxidative stress, and can inhibit inflammatory pathways, pivotal in delaying cellular senescence^19^. AMPK is a sentinel for energy stress caused by impaired mitochondrial activity in mitochondrial dysfunction. AMPK is upstream in signaling pathways linked to metabolic dysfunction, making it an integrative marker for early mitochondrial disturbances^20^.

p53 (tumor protein 53), a tumor suppressor protein, is vital for regulating the cell cycle, apoptosis, and DNA repair. In the context of senescence, p53 activation is associated with mitochondrial biogenesis and the suppression of dysfunctional cells. AMPK’s interaction with p53 enhances mitochondrial function and induces a metabolic shift favoring cellular repair and survival^21^. Its central role in stress response pathways and its ability to regulate other senescence markers make it indispensable for evaluating radiation effects. p21 (Cyclin-Dependent Kinase Inhibitor 1) is a downstream effector of p53 and is directly involved in cell cycle arrest during senescence. It inhibits cyclin-dependent kinases, preventing the cell cycle’s transition from the G1 to S phase. As a direct target of p53, p21 provides specific insight into the progression of senescence following mitochondrial impairment^22^. p16 (Cyclin-Dependent Kinase Inhibitor 2 A), a cyclin-dependent kinase inhibitor, is a hallmark of cellular senescence, contributing to the irreversible arrest of the cell cycle. Its expression reflects cumulative cellular stress and age-related decline in mitochondrial function. p16 is a widely recognized biomarker in aging and senescence research^23^.

Recently, delaying or preventing aging-related dysfunctions by eradicating cell senescence has been suggested as a more effective strategy than treating specific disorders^24^. Researchers are focusing on determining which substances, particularly natural ones included in a regular diet, can postpone the signs of aging. Curcumin, one such compound, is a hydrophobic polyphenol that dissolves in organic solvents. It exhibits various biological properties, such as anti-inflammatory, anti-tumor, and antioxidant activities^25^. Studies have demonstrated that curcumin can contribute to many biological processes, including defense mechanisms, caspases, cytokines, transcription factors, growth factors, and apoptosis^26–28^. Additionally, curcumin is a potentially beneficial anti-aging substance, reasonably priced, readily available, and easy to incorporate into the diet^29^. While curcumin has consistently shown promising results in various experimental studies, particularly its anti-aging properties, the evidence regarding its specific impact on cell senescence remains somewhat limited^30^.

Curcumin metabolizes in the liver, producing less active curcumin glucuronides, and is excreted from the body. It is poorly absorbed by intestinal cells and has low water solubility and stability^31^. Also, it has a limited passage through the blood-brain barrier^32^. Therefore, the potential therapeutic use of curcumin in clinical trials is limited. Curcumin was selected in this study due to its well-documented therapeutic efficacy in treating various diseases, including metabolic disorders^28^, cancer^33^, and neurodegenerative conditions^31^. Moreover, curcumin is generally recognized as safe and has demonstrated minimal toxicity, even at high doses, which makes it a promising candidate for therapeutic applications ^34,35^.

According to previous research, curcumin nanoformulations have increased solubility, absorption capacity, and bioavailability^36,37^. These nanoparticles provide more advantages in the therapeutic protocol, as enhanced bioavailability facilitates lower doses and reduces toxicity and side effects from long-term use. Tsai et al. reported that curcumin nanoparticles (CNPs) were distributed more efficiently to various rat organs, including the brain, than conventional curcumin formulations. Importantly, the mean residence time of CNPs in brain tissue was significantly prolonged. This suggests that CNPs could maintain effective concentrations in brain regions for extended periods, potentially enhancing their therapeutic potency in treating brain injuries. This extended retention highlights CNPs as a promising alternative to traditional curcumin, particularly for neurological applications^38^.

Based on this background, the current research aimed to evaluate the cellular toxicity and mitochondrial dysfunction during cell senescence in rat brain tissue exposed to γ radiation and the possible therapeutic role of curcumin nanoparticles (CNPs) in protecting against cell aging by evaluating oxidative status, neuroinflammatory responses, and signal pathways of genes mediated by this mechanism.

## Materials and methods

### Chemicals

The curcumin powder used in this study was procured from Sigma Aldrich, UK, and identified as 100% pure natural powder. All other chemicals utilized in this research were of analytical grade.

### Preparation of curcumin nanoparticles (CNPs)

Nano-sized curcumin powder was prepared by heating 0.1 g of curcumin with 100 mL of distilled water at 60 °C for one hour. The solution was then filtered to obtain a homogeneous mixture, and 2–3 drops of isopropanol were added to prevent further formation of free radicals. Finally, we exposed the solution to 15 kGy γ radiation to obtain CNPs^39^.

### Characterization of CNPs

The characterization processes involved the use of various techniques. The size and shape of CNPs were analyzed using a high-resolution transmission electron microscope (HRTEM, JEM-2100, JEOL, Japan). The surface properties and morphology of CNPs were determined using a scanning electron microscope (SEM) (ZEISS, EVO-MA10). The zeta potential and surface charge of CNPs were measured using a dynamic light scattering Zeta-sizer (Malvern 3000 HSa, UK).

### In vitro study

#### In vitro antioxidant activity of CNPs

In vitro, the antioxidant activity of CNPs was evaluated using the DPPH (1,1-diphenyl-2-picrylhydrazyl) radical scavenging method^40^. Different concentrations of the sample (curcumin, CNPs, and ascorbic acid as a reference antioxidant) in ethanol were prepared and ranged from 1.95 µg/mL to 1000 µg/mL. Next, 1.0 ml of DPPH solution (0.1 mM) was added to a 3.0 ml sample, shaken, and incubated at room temperature for 30 min. Absorbance (OD) at 517 nm was measured in triplicate, and the mean was used to calculate the percentage of the DPPH scavenging effect.

$${\text{DPPH scavenging effect }}\% {\text{ }}={\text{ A}}0 - {\text{A1}}/{\text{A}}0{\text{ }} \times {\text{ 1}}00$$

(where A0 = OD of the control reaction or 0 concentration and A1 = OD of the sample)

#### In vitro anti-inflammatory activity of CNPs

##### RBCs suspension preparation

Three mL of fresh rat whole blood was collected into heparinized tubes and centrifuged for 10 min at 3000 rpm. Discard the supernatant, then the packed red blood cells were resuspended using a volume of normal saline equivalent to that of the supernatant and reconstituted with isotonic buffer solution (10 mM sodium phosphate buffer, pH 7.4) as a 40% v/v suspension.

##### Hypotonicity-induced hemolysis

Different concentrations of curcumin and CNPs (100, 200, 400, 600, 800, and 1000 µg/mL) were prepared using distilled water to form hypotonic solutions, and the same concentrations were used to prepare isotonic solutions. Five mL of each concentration of isotonic and hypotonic solutions, 5 mL of indomethacin (200 µg/mL) as the reference standard, and 5 mL of distilled water (as the control) were placed in centrifuge tubes. A 0.1 mL erythrocyte suspension was added to each tube and mixed gently. The mixtures were incubated for 60 min at 37 °C and then centrifuged for 3 min at 1300 rpm. The absorbance (OD) of the hemoglobin content in the supernatant was measured at 540 nm. The readings were taken in triplicates, and the mean was used to calculate the percent inhibition of hemolysis^41^.

$${\text{Hemolysis inhibition }}\left( \% \right)\,=\,{\text{1 }} - {\text{ }}\left[ {\left( {{\text{A2 }} - {\text{ A1}}} \right)/\left( {{\text{A3 }} - {\text{ A1}}} \right)} \right]{\text{ }}*{\text{ 1}}00$$

Where A1 = OD of the test sample in the isotonic solution, A2 = OD of the test sample in the hypotonic solution, A3 = OD of the control sample in the hypotonic solution.

### Animals

Thirty-two male Wistar albino rats (120–150 g) were obtained from the National Cancer Institute, Egypt. The animals were acclimatized for seven days in a temperature-controlled environment (20–25 °C) with a 12-hour dark/light cycle and had free access to food and water. The research protocol was approved by the Research Ethics Committee, Faculty of Science, Ain Shams University, Cairo, Egypt (Code: ASU-SCI/BIOC/2023/5/4), and by the Research Ethics Committee for animal experimental studies at the NCRRT, Cairo, Egypt (Protocol Serial Number: P/55A/24). All methods were carried out according to relevant guidelines and regulations and are reported under the ARRIVE guidelines.

### Irradiation process

Rat head irradiation was performed at the National Center for Radiation Research and Technology, Cairo, Egypt, using an Indian ^60^Co Cell. Anesthesia was induced in the animals through an intraperitoneal injection of pentobarbital at a dose of 60 mg/kg^42^. The rats’ heads were then subjected to a single 10 Gy γ radiation dose^43^ at a rate of 0.6213 kGy/h for 58 s while the rest of their bodies were shielded with lead.

### Experimental design

**Fig. 1:**
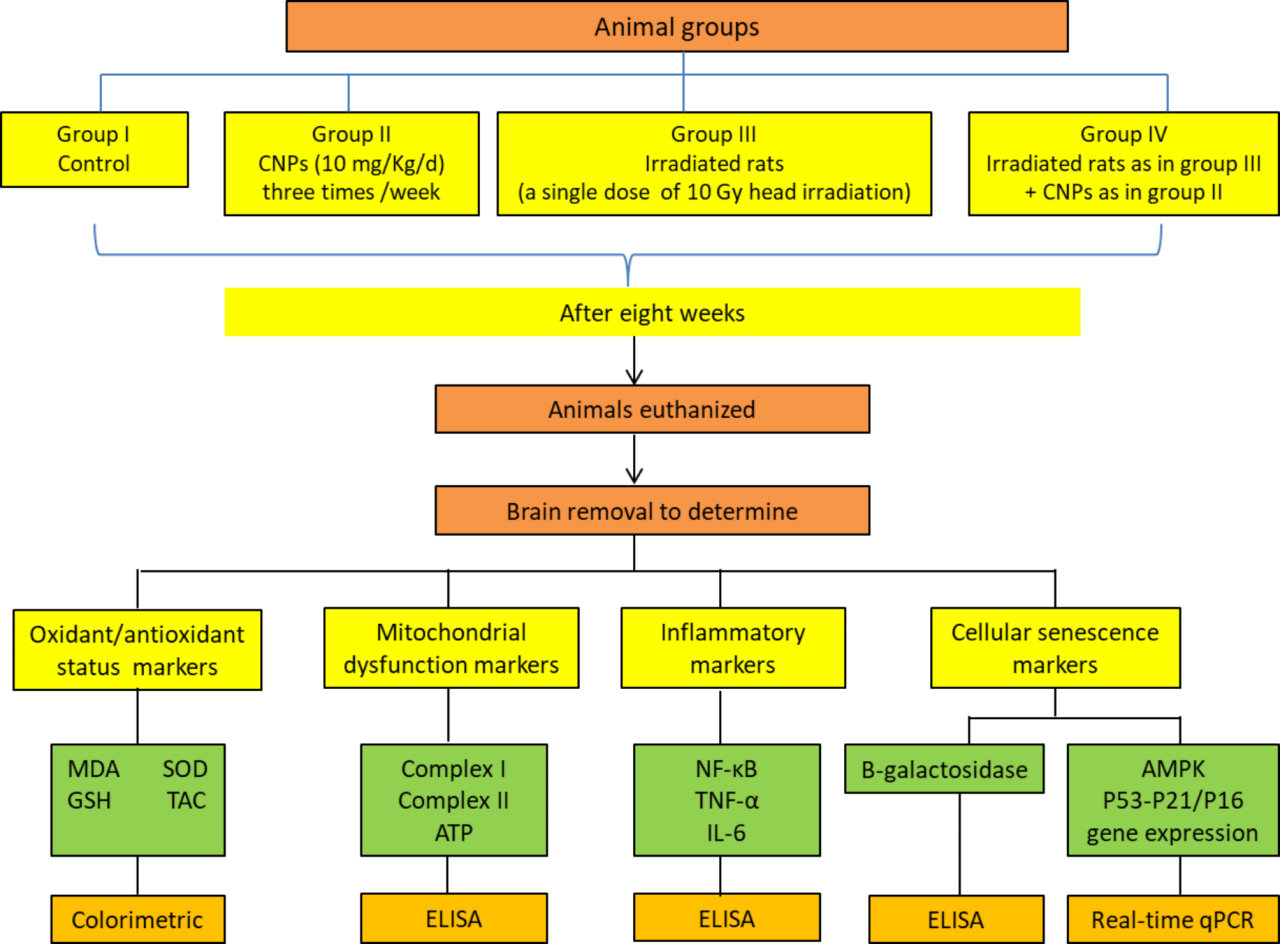
Experimental design.

The animals were divided into four groups, with eight rats in each group (*n* = 8) (Fig. 1). Group I (control group): Normal rats received no treatment. Group II (CNPs-treated group): Rats were orally administered CNPs (10 mg/kg/d in 1.0 mL distilled water) three times/week for eight weeks, each treatment separated by 1–2 days^44^. Group III (irradiated group): Rats were exposed to a single dose of 10 Gy head irradiation^43^. Group IV (irradiated rats + CNPs): Rats were exposed to a single dose of γ-radiation as in Group III, and thirty minutes after irradiation, they were orally administered CNPs (10 mg/kg/day in 1 mL distilled water) three times per week for eight weeks, as in Group II.

Eight weeks after exposure to radiation or treatment, the rats were anesthetized via intraperitoneal injection of pentobarbital (60 mg/kg) and euthanized by decapitation. The brains were quickly removed and placed in ice-cold Petri dishes. One part of the brain was homogenized in 10% PBS (phosphate-buffered saline, pH 7.4; 1.0 g brain tissue: 9.0 mL PBS) and centrifuged at 5000 rpm for 10 min at 4 °C. The resulting supernatant was stored at -80 °C until used for biochemical analysis. The brain homogenate was used to assess oxidant/antioxidant markers, inflammatory markers (NF-ĸB, IL-6, and TNF-α), and β-galactosidase levels. The second part of the brain tissue was used for quantitative real-time PCR analysis of AMPK, p53, p21, and p16 gene expression. The third part of the brain tissue was used for mitochondrial isolation and determination of complex I and complex II activity.

### Biochemical analysis

#### Effect of CNPs on oxidant/antioxidant status

Malondialdehyde (MDA), a lipid peroxidation marker, total antioxidant capacity (TAC), and reduced glutathione (GSH) levels were measured in brain tissue homogenate using Bio-Diagnostics colorimetric kits (CAT. NO. MD 25 29, TA 25 13, and GR 25 10, Bio-Diagnostics Co., Egypt), respectively, following the manufacturer’s guidelines. The activity of superoxide dismutase (SOD) was measured using the SOD Kit (CAT. NO. SD 25 21) from Bio-Diagnostics Co., Egypt, as described by the manufacturer.

### Effect of CNPs on biochemical markers of mitochondrial dysfunction

Brain mitochondria were isolated using a mitochondria isolation kit (CAT. NO. ab288084) from Abcam, UK. Briefly, brain tissue was rinsed twice in cold mitochondria isolation buffer and then homogenized in mitochondria isolation buffer with a protease inhibitor cocktail (CAT. NO. ab271306, Abcam, UK) added to prevent protein degradation at a ratio of 1:5 (W/V). The homogenate was centrifuged at 600 × g for 10 min, followed by centrifugation at 7000 × g for 10 min at 4 °C. The final pellet was used according to the manufacturer’s instructions. Complex I (NADH: ubiquinone oxidoreductase) and complex II (succinate dehydrogenase) activities were measured in the brain mitochondria, and ATP (the primary energy currency of living systems) levels were estimated in brain tissues using colorimetric assay kits (CAT. NO. K968-10, K660-100, and K354-100, respectively) from BioVision Inc., USA, following the manufacturer’s instructions.

### Effect of CNPs on inflammatory markers

Nuclear factor kappa B (NF-ĸB), the pro-inflammatory cytokines interleukin 6 (IL-6), and tumor necrosis factor-alpha (TNF-α) were measured in brain tissues using rat-specific ELISA kits (CAT. NO. MBS2505513, R6000B, and E11987r) from MyBioSource, Quantikine, and CUSABIO Inc., USA, respectively, according to the manufacturers’ instructions.

### Effect of CNPs on biochemical markers of cellular senescence

The β-galactosidase level was estimated using a rat-specific ELISA kit (CAT. NO. MBS261987) from MyBioSource Inc., USA, according to the manufacturer’s instructions. Tissue homogenate was processed for RNA extraction, followed by reverse transcription (for cDNA synthesis) and quantitative real-time PCR to quantify AMP-activated protein kinase (AMPK) and aging-related pathway (p53/p21/p16) gene expression using kits from Thermo Fisher Scientific, USA. The specific primer sequences for the examined genes and β-actin, used as the housekeeping (reference) gene, are shown in Table 1.

**Table 1:** Primer sequence of studied genes.

Gene	Primer sequence
AMPK	Forward: 5′-ATCCGCAGAGAGATCCAGAA-3′ Reverse: 3′-CGTCGACTCTCCTTTTCGTC-5′
p53	Forward: 5′-CTACTAAGGTCGTGAGACGCTGCC - 3′ Reverse: 3′-TCAGCATACAGGTTTCCTTCCACC-5′
p21	Forward: 5′-GCT TCATGCCAG CTACTTCC-3′ Reverse: 3′-CCC TTCAAAGTGCCA TCT GT-5′
p16	Forward: 5′-CTCACCATGGATGATGATATCGC- 3′ Reverse: 3′- AGGAATCCTTCTGACCCATGC- 5′
Beta-actin (reference gene)	Forward: 5′-AAGATCCTGACCGAGCGTGG- 3′ Reverse: 3′-CAGCACTGTGTTGGCATAGAGG-5′

### Statistical analysis

All experimental data were analyzed using the Statistical Package for the Social Sciences (SPSS, version 20). The results are presented as mean ± SE, and the significance between groups was assessed using a one-way analysis of variance (ANOVA). Differences between groups were considered significant when the p-value was ˂ 0.05, and comparisons between groups were evaluated using Tukey’s post hoc test.

## Results

### Characterization of CNPs

**Fig. 2:**
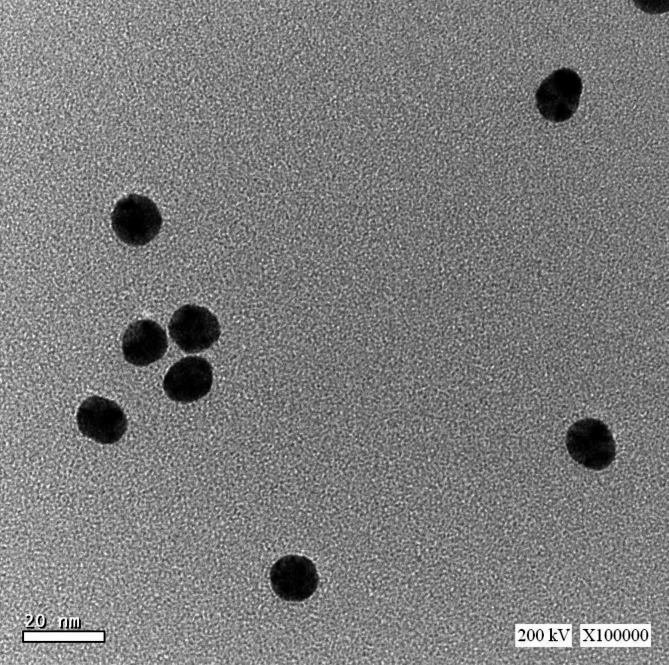
Characterization of CNPs. Particle size determination of CNPs by transmission electron microscope (TEM).

**Fig. 3:**
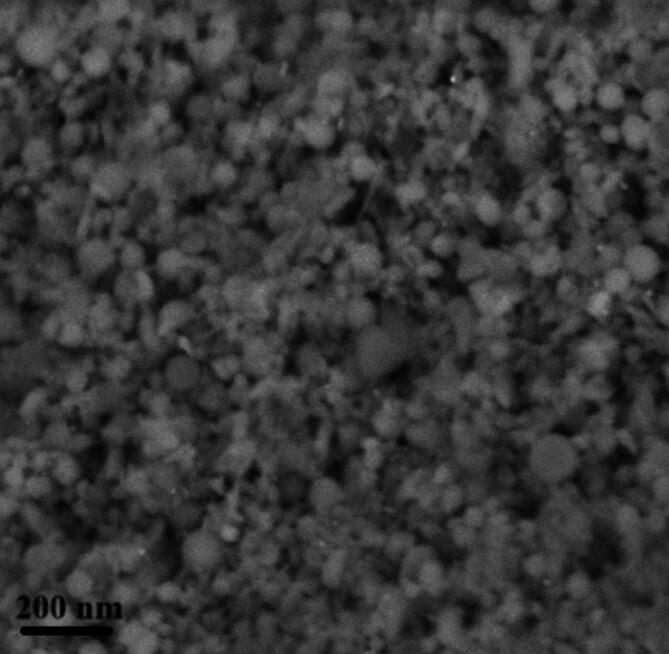
Characterization of CNPs. Surface properties and shape of the CNPs by scanning electron microscope (SEM).

**Fig. 4:**
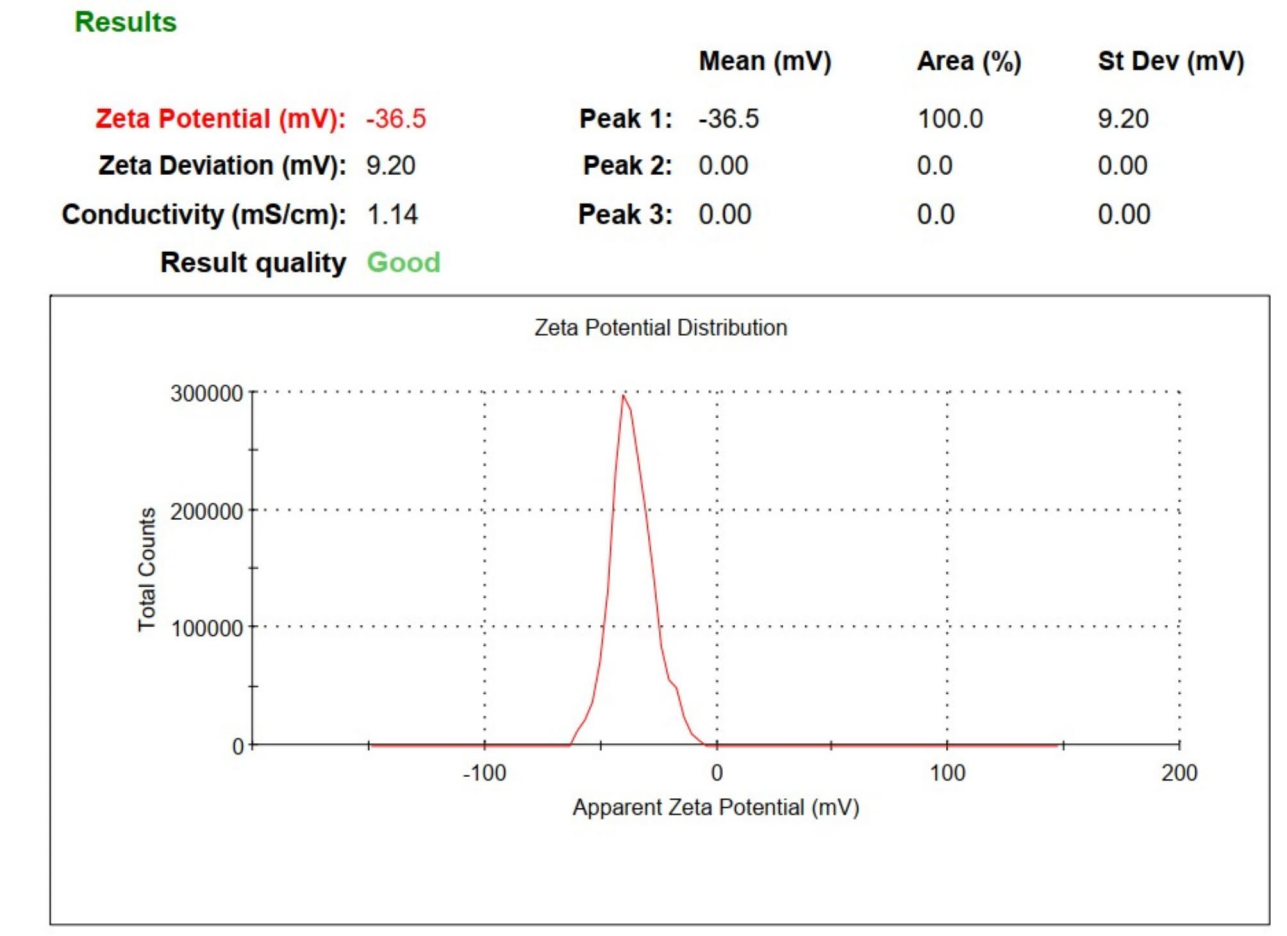
Characterization of CNPs. the zeta potential of the synthesized CNPs using a dynamic light scattering Zeta-sizer (zeta potential = -36.5 mV).

The HR-TEM image of the prepared CNPs revealed that the particles were semi-spherical (Fig. 2), with sizes ranging from 15.2 nm to 18 nm and an average diameter of 17.2 ± 0.5 nm. As investigated using SEM, the surface properties and morphology of the CNPs showed consistent surfaces with a uniform appearance (Fig. 3). Additionally, the CNPs appeared as bright, spherical particles that were well-defined and closely packed. These results confirm that the prepared CNPs were predominantly spherical and well-separated. As shown in Fig. 4, the zeta potential of the synthesized CNPs was analyzed at pH 7.2. The results of this study indicated that the surface zeta potential of the CNPs remained negative under these conditions. Furthermore, the zeta potential of the preparation in a neutral medium (pH 7.2) was recorded as -36.5 mV.

### In vitro study of the antioxidant and anti-inflammatory activities of CNPs

Table 2 presents the results of the antioxidant properties of curcumin and CNPs at various concentrations (1.95–1000 µg/ml) compared to the standard ascorbic acid using the DPPH method. The results demonstrated that curcumin and CNPs exhibited comparable antioxidant activity to ascorbic acid at equivalent concentrations.

**Table 2:** In vitro antioxidant activity of different concentrations of curcumin and CNPs compared to ascorbic acid as a reference antioxidant.

Concentration (µg/ml)	Curcumin	CNPs	Ascorbic acid
	DPPH radical scavenging effect (%) *		
1.95	16.5	2.9	37.9
3.9	41.9	17.5	64.2
7.81	67.7	35.2	78.0
15.62	94.2	58.3	86.3
31.25	95.2	70.6	88.2
62.5	95.5	90.0	88.9
125	95.7	96.7	90.6
250	95.8	97.0	91.1
500	96.7	96.9	93.5
1000	96.3	97.0	94.2

*Absorbance (OD) at 517 nm was measured in triplicate, and the mean was used to calculate the percentage of the DPPH scavenging effect.

Table 3 illustrates the hemolysis inhibition percentage of rat red blood cell (RBC) membranes, which measured the anti-inflammatory mechanism of curcumin and CNPs, compared with the standard indomethacin at 100–1000 µg/ml. The results showed that the hemolysis inhibition percentage of curcumin and CNPs increased proportionally with their concentrations, closely matching the effect of indomethacin. These findings suggest that curcumin and CNPs are capable of protecting RBC membranes against hypotonicity-induced hemolysis.

**Table 3:** In vitro anti-inflammatory activity of different concentrations of curcumin and CNPs compared to the anti-inflammatory indomethacin reference drug.

Concentration (µg/ml)	Curcumin	CNPs	Indomethacin
	RBC hemolysis inhibition (%)*		
100	87.2	80.6	93.3
200	90.1	84.0	94.8
400	92.4	87.7	96.0
600	95.0	90.0	97.9
800	97.9	93.5	98.9
1000	99.6	97.1	99.5

*Absorbance (OD) at 540 nm was measured in triplicate, and the mean was used to calculate the percentage of inhibition of hemolysis.

### Biochemical analysis

The current study demonstrated that exposing rats to 10 Gy head irradiation induced oxidative stress, mitochondrial dysfunction, inflammation, and cellular senescence in brain tissue. However, administration of CNPs effectively mitigated these damaging effects (Tables 4, 5 and 6; Fig. 4).

**Table 4:** Effect of radiation and curcumin nanoparticles on brain levels of malondialdehyde (MDA), superoxide dismutase (SOD) activity, reduced glutathione (GSH) content, and total antioxidant capacity (TAC) in experimental groups.

Groups	Parameters			
	MDA (nmol/mg)	SOD (U/mg protein)	GSH (mmol/mg)	TAC (mmol/mg)
Group I (control)	70.86 ^a^ ± 3.12	119.46 ^a^ ±1.49	84.80 ^a^ ± 1.52	49.86 ^a^ ± 1.76
Group II (CNPs)	67.52 ^a^ ± 2.17 (− 4.7)	124.97 ^a^ ± 0.92 (4.6)	87.01 ^a^ ± 1.35 (2.6)	55.08 ^b^ ± 1.32 (10.5)
Group III (irradiated rats)	218.90 ^b^ ± 5.08 (208.9)	56.75 ^c^ ± 1.84 (− 52.5)	31.75 ^b^ ± 1.06 (− 62.6)	16.63 ^c^ ± 1.55 (− 66.6)
Group IV (irradiated rats + CNPs)	93.70 ^c^ ±2.05 (32.2)	111.27 ^a^ ± 1.54 (− 6.9)	70.17 ^c^ ± 1.49 (− 17.3)	41.98 ^d^ ± 1.69 (− 15.8)

Data expressed as mean ± S.E. Different letters in the same column represent statistically significant values (P value ˂ 0.05), while the same letters represent statistically non-significant values. Values in brackets represent the % change compared to the normal control group. (/mg) = (/milligram brain tissue).

**Table 5:** Effect of radiation and curcumin nanoparticles on complex I (NADH: ubiquinone oxidoreductase), complex II (succinate dehydrogenase) activities, and ATP level in brain tissues of experimental groups.

Groups	Parameters		
	Complex I (NADH: ubiquinone oxidoreductase) (U/mg protein)	Complex II (succinate dehydrogenase) (U/mg protein)	ATP (µmol/g brain tissue)
Group I (control)	27.56 ^a^ ± 1.76	42.30 ^a^ ± 2.59	137.32 ^a^ ± 2.61
Group II (CNPs)	29.80 ^a^ ± 1.30 (8.1)	45.53 ^a^ ± 1.02 (7.6)	140.92 ^a^ ± 1.88 (2.6)
Group III (irradiated rats)	10.23 ^b^ ± 0.56 (− 62.9)	23.76 ^b^ ± 1.07 (-43.8)	66.51 ^b^ ± 1.32 (− 51.6)
Group IV (irradiated rats + CNPs)	21.43 ^c^ ± 1.08 (− 22.2)	39.72 ^a^ ± 0.94 (− 6.1)	119.68 ^c^ ± 1.20 (− 12.8)

Data expressed as mean ± S.E. Different letters in the same column represent statistically significant values (P value ˂ 0.05), while the same letters represent statistically non-significant values. Values in brackets represent the % change compared to the normal control group.

**Table 6:** Effect of radiation and curcumin nanoparticles on brain levels of tumor necrosis factor-alpha (TNF-α), interleukin-6 (IL-6), and nuclear factor kappa B (NF-ĸB) in experimental groups.

Groups	Parameters		
	TNF-α (pg/mg)	IL-6 (pg/mg)	NF-ĸB (pg/mg)
Group I (control)	19.86 ^a^ ± 0.92	39.28 ^a^ ± 1.64	108.06 ^a^ ± 1.69
Group II (CNPs)	15.91 ^a^ ± 0.91	36.60 ^a^ ± 0.92	106.88 ^a^ ± 1.52
Group III (irradiated rats)	83.00 ^b^ ± 3.01	120.65 ^b^ ± 2.15	323.83 ^b^ ± 8.58
Group IV (irradiated rats + CNPs)	43.93 ^c^ ± 2.55	60.50 ^c^ ± 2.80	145.53 ^c^ ± 2.94

Data expressed as mean ± S.E. Different letters in the same column represent statistically significant values (P value ˂ 0.05), while the same letters represent statistically non-significant values. (/mg) = (/milligram brain tissue).

### Effect of CNPs on oxidant/antioxidant status

The results in Table 4 indicated that rat head irradiation at a dose of 10 Gy elicited oxidative stress in brain tissue. These results were evidenced by a significant increase (*P* < 0.05) in MDA levels, accompanied by a significant decrease (*P* < 0.05) in SOD activity, GSH content, and TAC levels compared to the control group.

In Group II, administration of CNPs (10 mg/kg) three times per week for eight weeks in normal rats resulted in a significant increase in brain TAC levels, with no significant changes observed in SOD activity, MDA levels, or GSH content compared to the control group.

However, oral administration of CNPs (10 mg/kg) for eight weeks post-irradiation (Group IV) induced a significant decrease (*P* < 0.05) in MDA levels and a significant increase (*P* < 0.05) in SOD activity, GSH content, and TAC levels compared to the corresponding values in the irradiated rat group (Group III). Notably, SOD activity was restored to control levels following CNP treatment.

### Effect of CNPs on biochemical markers of mitochondrial dysfunction

Exposure of rats to head irradiation disrupted brain mitochondrial function. This impact was evidenced by a significant depletion (*P* < 0.05) in the activities of mitochondrial complexes I and II, as well as ATP production, compared to the control group (Table 5). When normal rats were treated with CNPs, no significant changes were observed in these parameters compared to the control group. However, rats administered CNPs post-irradiation (Group IV) showed a significant increase (*P* < 0.05) in the activities of mitochondrial complexes I and II and ATP production compared to the irradiated group (Group III). Notably, the activity of complex II was restored to control values following CNPs treatment.

### Effect of CNPs on inflammatory markers

Table 6 shows that rats exposed to head irradiation exhibited significantly elevated (*P* < 0.05) levels of pro-inflammatory cytokines (IL-6 and TNF-α) and NF-κB in brain tissues compared to the control group. Conversely, oral administration of CNPs post-γ-radiation exposure (Group IV) significantly (*P* < 0.05) attenuated the effects of irradiation, resulting in a reduction in brain inflammatory markers (TNF-α, IL-6, and NF-κB) compared to the irradiated group (Group III).

### Effect of CNPs on biochemical markers of cellular senescence

**Fig. 5:**
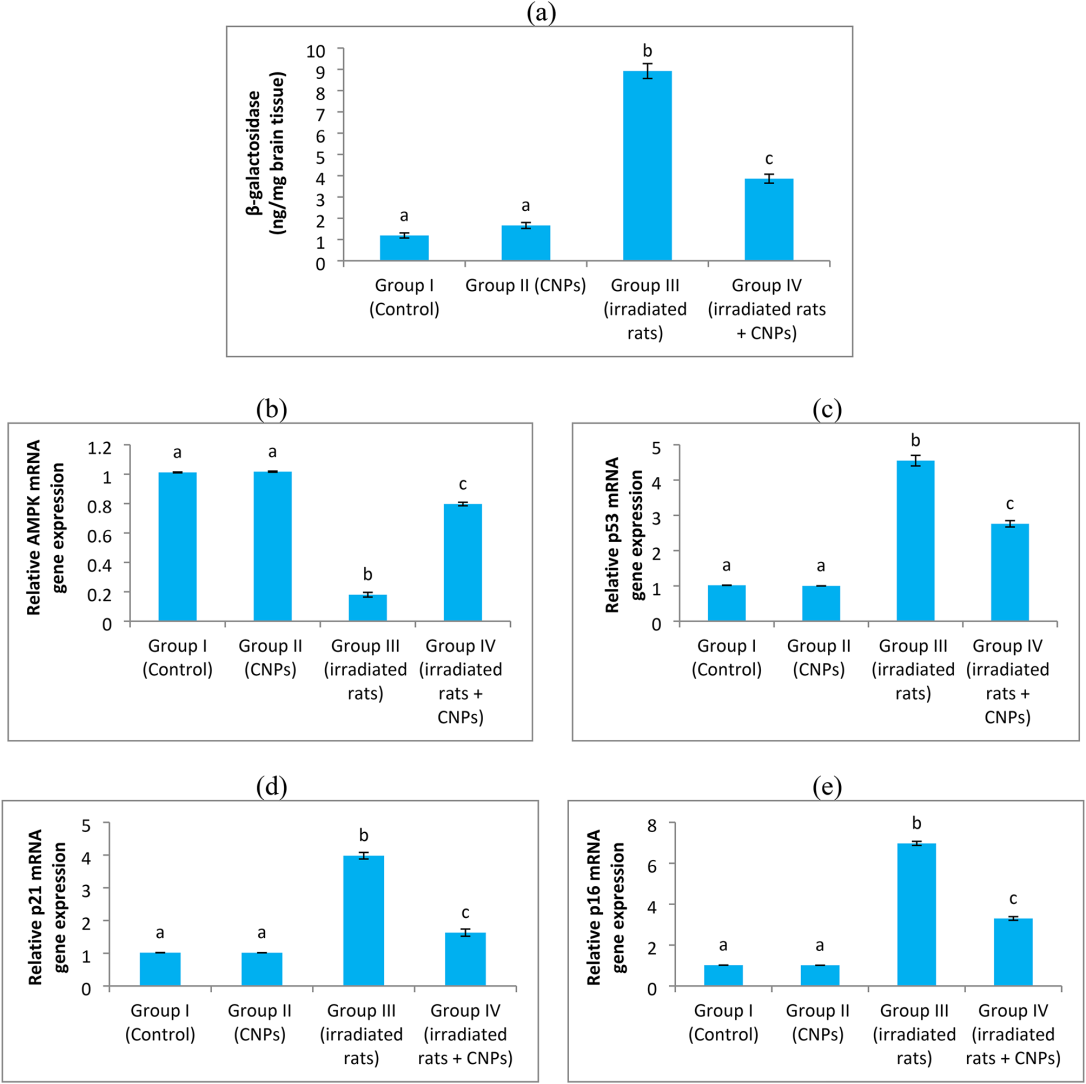
Effect of radiation and curcumin nanoparticles on (**a**) β-galactosidase activity and mRNA gene expression levels of (**b**) adenosine 5ʹmonophoshate-activated protein kinase (AMPK), (**c**) p53, (**d**) p21, and (**e**) p16. Data expressed as mean ± S.E. Different letters represent statistically significant values (P value ˂ 0.05), while the same letters represent statistically non-significant values.

After exposure to head irradiation, rats exhibited signs of cellular senescence in brain tissue. These signs were evidenced by a significant increase (*P* < 0.05) in β-galactosidase levels and the gene expression of p53, p16, and p21, along with a concurrent significant decrease in AMPK mRNA expression compared to the control group. Conversely, oral administration of CNPs to irradiated rats effectively modulated these indicators of cellular senescence, as shown in Fig. 5.

## Discussion

Senescence is a complex biological process characterized by an exponential increase in chronic illnesses, ultimately leading to systemic dysfunction. Mitochondrial dysfunction is one of the primary hallmarks of aging and is closely linked to cellular senescence^4^. It is strongly associated with failures in cellular energy production and maintenance, as free radicals and reactive oxygen species (ROS) continuously assault cells throughout their lifespan^45^.

Under physiological conditions, respiratory and metabolic processes produce ROS as byproducts of redox reactions^46^. However, excessive ROS production can occur due to various internal and external stimuli, leading to oxidative stress. Oxidative stress induces several pathogenic alterations in cells, including telomere shortening, DNA damage, lipid peroxidation, protein oxidative modification, and mitochondrial dysfunction, all of which contribute to cellular senescence and eventual cell death.

Furthermore, oxidative stress is a key driver in the development of a range of aging-related diseases, such as retinal disorders, neurodegenerative diseases, and cardiovascular diseases^8^. Notably, mitochondria and oxidative stress are intricately linked. Oxidative stress can result in mitochondrial dysfunction by adversely affecting mitochondrial dynamics, shape, and membrane potential, which in turn exacerbates oxidative stress.

The release of ROS by γ-radiation induces oxidative stress, disrupting the balance between oxidants and antioxidants in various cells, ultimately leading to cellular senescence and cell death^47^. Free radicals generated and accumulated within stressed cells overwhelm the body’s natural antioxidant defenses, causing damage to cellular components such as proteins, nucleic acids, and lipids^48^. Malondialdehyde (MDA), a byproduct of lipid peroxidation, is the most widely used biomarker of oxidative stress. MDA compromises ion transport and increases cell membrane permeability. This disruption of ion balance within the cell further impairs enzyme activity^49^.

The current study demonstrated that exposing rats to head irradiation induced the production of free radicals and oxidative stress. This effect was evidenced by a significant increase in lipid peroxidation (MDA) levels, accompanied by a concomitant significant decrease in antioxidant biomarkers, including SOD activity and GSH levels, compared to the control group. Consistent with our findings, previous studies^50,51^ have shown that radiation energy can cause the radiolysis of water in tissues and cells, leading to the rapid generation of reactive nitrogen species and ROS. Following the initial cellular damage caused by radiation energy, the oxidation/reduction system generates free radicals a few hours post-exposure.

In agreement with this study, Zhu et al.^52^ reported that exposure to ionizing radiation results in lower levels of reduced glutathione (GSH), catalase, glutathione transferase, and SOD activity compared to controls. Furthermore, the present study demonstrated that ROS generated by γ-radiation exposure reduced TAC levels during the ROS-neutralization process by targeting antioxidant molecules.

Radiation exposure causes mitochondrial dysfunction by increasing ROS levels, disrupting the electron transport chain, and inducing oxidative stress. Senescent cells accumulate defective mitochondria, further enhancing ROS generation and promoting the senescence-associated secretory phenotype (SASP)^53^. In this study, the ability of CNPs to reduce ROS levels in rats exposed to γ-radiation was evident from the decreased concentrations of the lipid peroxidation marker (MDA), increased GSH and TAC levels, and normalized antioxidant SOD activity. These results indicate that CNPs alleviated oxidative stress in γ-irradiated rats by scavenging ROS. Previous research has shown that persistently high ROS levels adversely affect mitochondrial protective mechanisms, induce mitochondrial DNA alterations, damage the mitochondrial electron transport chain, and modify membrane permeability^54^.

Consequently, the antioxidant effects, evidenced by decreased MDA levels and restored SOD activity^55^, are likely the mechanism by which improved mitochondrial function is mediated in irradiated rats treated with CNPs. Our results are consistent with other research demonstrating that curcumin can reduce oxidative stress by directly scavenging ROS and upregulating antioxidant enzymes^56^. Additionally, curcumin exhibits properties that help preserve mitochondrial integrity^57^. A previous study reported that oral nano-curcumin modulates mitochondrial function and oxidative stress, reducing neurological disorders in a rat model of chronic Gulf War disease^44^. Furthermore, in the present study, the in vitro antioxidant activity results indicated that CNPs exhibited antioxidant activity comparable to native curcumin and ascorbic acid at equivalent concentrations.

The primary function of mitochondria is to produce ATP through a sequence of proteins known as the multimeric enzyme complexes of the electron transport chain (ETC), which facilitate electron transport from NADH and FADH₂. At the beginning of the ETC, complex I (NADH: ubiquinone oxidoreductase) and complex II (succinate: ubiquinone oxidoreductase) reduce the primary electron acceptor, providing entry points for electrons from NADH and FADH₂. At the terminus of the chain, water is formed when electrons combine with protons and molecular oxygen after passing through complexes III and IV^58^. Under conditions of high metabolic stress, the dissipation of the electrochemical proton gradient reduces ATP production, resulting in mitochondrial ultrastructural anomalies, loss of respiratory chain function and mitochondrial enzymes, and increased ROS formation, particularly in neurons^59^. Mitochondria also play critical roles in detecting oxidative stress and coordinating protective mechanisms to mitigate excessive ROS generation and prevent potential mutagenesis, including the formation of oxidatively damaged DNA^60^.

Mitochondria are well-recognized as intracellular targets for radiation. Mitochondrial DNA (mtDNA) is more susceptible to oxidative stress than nuclear DNA due to its lack of histone protection and an efficient DNA repair system. This vulnerability leads to disruptions in the oxidative phosphorylation pathway, which is critical for ATP production^61^. In the present study, rats exposed to head irradiation exhibited a significant reduction in the activity of mitochondrial complex proteins I and II and ATP production levels compared to the control group, indicating hypoactive mitochondria. These findings align with earlier studies demonstrating similar mitochondrial dysfunction following radiation exposure^62,63^.

This study highlights the ability of CNPs to enhance mitochondrial function in the brains of γ-irradiated rats, as demonstrated by several key findings. Irradiated rats treated with CNPs exhibited improved mitochondrial activity, evidenced by increased activity of mitochondrial complexes I and II and elevated levels of ATP synthesis. Notably, the activity of complex II was restored to control levels following CNP treatment. Sood et al. reported that curcumin administration restored the activity of mitochondrial complexes I, II, and IV in the brain tissues of aluminum-treated rats^64^. Consistent with our results, a previous study demonstrated that curcumin enhances the activity of mitochondrial ETC complex enzymes and antioxidant enzymes, providing neuroprotective and mitochondrial protection against the effects of rotenone withdrawal^65^.

Mitochondrial complex enzymes I, II, III, and IV and the potential of the mitochondrial membrane can be preserved by natural curcumin and its derivatives, thereby safeguarding against mitochondrial dysfunctions^66^. Furthermore, in an animal model exposed to chemicals and stress associated with Gulf War disease, two months of nano-curcumin therapy significantly reduced the impairment of mitochondrial complexes II and IV^44^.

Under normal physiological conditions, the brain balances between pro- and anti-inflammatory mediators^67^. However, this balance shifts toward a pro-inflammatory state with aging, a phenomenon observed following radiation exposure^61^. This suggests that radiation-induced neuroinflammation could be a contributing factor to brain aging. Consistent with previous studies^68,69^, our findings demonstrated that rats exposed to head irradiation exhibited significantly elevated levels of NF-κB and pro-inflammatory mediators, including IL-6 and TNF-α, in brain tissues. Ibragimova et al. reported that ionizing radiation activates microglial cells in brain tissue, stimulating the production of cytokines, promoting inflammation, and inducing metabolic changes. These processes are key drivers of radiation-induced brain aging and increase the risk of neurodegenerative diseases. Additionally, NF-κB activation in irradiated rat tissues has been shown to upregulate the mRNA expression of pro-inflammatory cytokines such as IL-1α, IL-1β, IL-6, and TNF-α^2^.

Treatment of rats with CNPs following γ-radiation exposure mitigated the adverse effects of irradiation, significantly reducing the levels of brain inflammatory markers, including NF-κB, TNF-α, and IL-6, compared to the irradiated group. Previous studies^70,71^ have demonstrated that the anti-inflammatory effects of curcumin are largely attributed to its ability to suppress NF-κB activity and decrease TNF-α levels, as TNF-α is a potent activator of the NF-κB pathway.

Additionally, curcumin’s antioxidant properties have been shown to influence NF-κB activation, thereby reducing inflammation and cellular damage^72,73^. By inhibiting NF-κB, curcumin helps prevent oxidative stress-induced cellular damage, a critical factor in aging processes. This dual role of curcumin, combining antioxidant and anti-inflammatory effects, contributes to its ability to protect against cellular damage, promote immune system resilience, and delay the onset of aging-related symptoms. Thus, curcumin serves as a pivotal biochemical agent for reversing inflammation and enhancing overall health.

Senescent cells are identified using a combination of markers that can differentiate them from quiescent or differentiated cells and highlight their state of stable growth arrest. Senescence-associated β-galactosidase (SA-β-gal), a lysosomal enzyme, remains the most widely used biomarker for detecting senescent cells in fresh and cultured tissue samples. Cano et al. reported that activating enzymes such as AMPK, recognized as a putative longevity factor, is a promising avenue for effective anti-aging strategies. AMPK also serves as a key marker of cellular aging^74^. Furthermore, tumor suppressor pathways regulated by p53 play a pivotal role in senescence, with the cyclin-dependent kinase inhibitors p21 and p16 being critical components. These inhibitors commonly accumulate in senescent cells and are sufficient to initiate and sustain the senescence-associated growth arrest. In both tissues and cultured cells, elevated levels of p21 and p16 are hallmark indicators of cellular senescence^10^.

Consistent with previous studies^61,75^, our findings demonstrate that irradiated rats exhibited clear signs of cellular senescence in their brain tissues. These indicators included a significant increase in β-galactosidase activity and upregulated gene expression levels of p53, p21, and p16, alongside a concurrent significant decrease in AMPK mRNA expression compared to the control group.

Radiation exposure induces oxidative stress and DNA damage, activating the tumor suppressor protein p53 as part of the cellular stress response. This activation leads to transcriptional regulation of multiple downstream targets, including p21, which enforces cell cycle arrest by inhibiting cyclin-dependent kinases^1^. This mechanism is critical for allowing DNA repair or promoting senescence to prevent the propagation of damaged cells. In this study, the upregulation of p53 and p21 observed in irradiated rats underscores their roles as markers of radiation-induced cellular senescence and the potential of curcumin nanoparticles in modulating these pathways. By mitigating oxidative stress and preserving mitochondrial integrity, curcumin nanoparticles may reduce the sustained activation of p53-p21 signaling, thereby alleviating mitochondrial dysfunction and delaying senescence progression. This finding is consistent with prior studies demonstrating that radiation-induced senescence is mediated by persistent DNA damage and stress signaling, through the p53-p21 axis^1,2^.

A recent study^5^ highlighted that senescence is characterized by the upregulation of senescence-associated β-galactosidase and tumor suppressor genes such as p53, p21, and p16, widely recognized as key markers of cellular senescence. Supporting these findings, Kumari and Jat^76^ reported that the upregulation of p21WAF1/CIP1 occurs via p53-dependent/non-INK/ARF Locus pathways, whereas p16INK4A is regulated via non-p53-dependent/INK/ARF Locus pathways, both of which are indicative of cellular senescence.

Additionally, Kim et al. demonstrated that mitochondrial dysfunction leads to increased p21 expression, inhibition of cellular proliferation, and cell cycle disruption through the regulation of p53. Stressors such as radiation exposure lower cellular ATP levels and elevate AMPK expression, a critical regulator of cellular energy homeostasis. Interestingly, persistent activation of AMPK has been shown to accelerate p53-mediated cellular senescence^1^.

In the context of mitochondrial dysfunction, p53 plays a role in regulating mitochondrial biogenesis and maintaining mitochondrial integrity by influencing pathways such as PGC-1α (peroxisome proliferator-activated receptor-gamma coactivator 1-alpha)^77^. As a downstream effector of p53, p21 enforces cell cycle arrest in response to stress by inhibiting cyclin-dependent kinases. This prevents damaged cells from proliferating^22^.

The senescence pathway, particularly the p53-p21 signaling pathway, is one of the most extensively studied mechanisms underlying cell aging induced by ionizing radiation^2^. The genes p53 and p21 play critical roles in a conserved route of cell growth inhibition and are key initiators of cellular senescence^22,78^. Another significant pathway is the p16/pRB pathway, which is crucial for maintaining senescence^79^. Activated p53 induces senescence by triggering p21, leading to G1 cell cycle arrest^76^, whereas p16-mediated senescence, which is regulated independently of p53, inhibits Rb phosphorylation, thereby preventing the transition from the G1 to S phase of the cell cycle^80^.

In the present study, CNPs supplementation in γ-irradiated rats significantly reduced the levels of β-galactosidase and the gene expression of p53, p21, and p16 while concurrently increasing AMPK mRNA expression compared to untreated irradiated rats. These findings suggest that CNPs may act as a potent anti-senescence agent. Supporting our results, Wong et al. demonstrated that curcumin induces glioblastoma cells to undergo G2/M arrest by upregulating p53 and p21 expression in a concentration- and time-dependent manner^81^. Similarly, an earlier study showed that in senescent cardiac cells induced by D-galactose, the number of cells positive for senescence-associated β-galactosidase, p16, and p53 significantly increased. However, this effect was reversed in a dose-dependent manner by curcumin treatment^82^. Moreover, previous studies have highlighted the neuroprotective effects of native curcumin in various diseases by activating the AMPK pathway^83,84^. A recent study further demonstrated that curcumin is an AMPK activator, enhancing AMPK phosphorylation to counteract cellular aging and stress responses^73^.

## Conclusion

Our study concluded that oral administration of CNPs (10 mg/kg) in γ-irradiated rats attenuates brain mitochondrial dysfunction and cellular senescence by enhancing the oxidant/antioxidant status, increasing the activity of mitochondrial complexes I and II, and boosting ATP production. Additionally, it reduces the levels of brain inflammatory mediators (TNF-α, IL-6, and NF-κB) and β-galactosidase while downregulating the gene expression of senescence pathway activators (p53-p21/p16 signaling pathway) and simultaneously elevating AMPK mRNA expression levels. According to our findings, CNPs act as an anti-senescence agent, delaying cellular senescence and enhancing mitochondrial function.

## Data Availability

All data for this study are included in this published article.
